# In Vitro Tissue Culture in *Brachypodium*: Applications and Challenges

**DOI:** 10.3390/ijms21031037

**Published:** 2020-02-04

**Authors:** Alexander Betekhtin, Karolina Hus, Magdalena Rojek-Jelonek, Ewa Kurczynska, Candida Nibau, John H. Doonan, Robert Hasterok

**Affiliations:** 1 Institute of Biology, Biotechnology and Environmental Protection, Faculty of Natural Sciences, University of Silesia in Katowice, 28 Jagiellonska Street, 40-032 Katowice, Poland; karolinazubrzycka92@gmail.com (K.H.); magdalena.rojek@us.edu.pl (M.R.-J.); ewa.kurczynska@us.edu.pl (E.K.); robert.hasterok@us.edu.pl (R.H.); 2 National Plant Phenomics Centre, IBERS, Aberystwyth University, Aberystwyth SY23 3EE, UK; csn@aber.ac.uk (C.N.); jhd2@aber.ac.uk (J.H.D.)

**Keywords:** *Agrobacterium*, *Brachypodium distachyon*, *Brachypodium* species, cell wall, genes, model plant, somatic embryogenesis, transformation

## Abstract

*Brachypodium distachyon* has become an excellent model for plant breeding and bioenergy grasses that permits many fundamental questions in grass biology to be addressed. One of the constraints to performing research in many grasses has been the difficulty with which they can be genetically transformed and the generally low frequency of such transformations. In this review, we discuss the contribution that transformation techniques have made in *Brachypodium* biology as well as how *Brachypodium* could be used to determine the factors that might contribute to transformation efficiency. In particular, we highlight the latest research on the mechanisms that govern the gradual loss of embryogenic potential in a tissue culture and propose using *B. distachyon* as a model for other recalcitrant monocots.

## 1. Introduction

The use of model organisms can greatly facilitate and accelerate the analyses of complex biological processes. Over the last three decades, *Arabidopsis thaliana* (Arabidopsis) has served as a model system to study the mechanisms that control plant development, ecology, evolution, physiology, cell biology and genetics [1]. Despite many excellent features that facilitate using it for research and its similarity to many crops, the usefulness of Arabidopsis as a model is limited in an analysis of monocot-specific processes. Among the monocot plants, grasses represent the key crop group and provide the majority of food calories globally. Various crop species have been proposed as models for grass biology—initially maize, then barley and, more recently, wheat. However, the large size of individuals as well as their large genome size and the long generation time can be demanding on growth facilities, and there is often limited access to the germplasm [2].

*Brachypodium distachyon* is a small rapidly cycling wild member of the Pooideae subfamily. Its natural range includes the Mediterranean basin, Middle East, south-west Asia and north-east Africa [3,4,5]. As a result of naturalisation, populations are also found in North and South America, Australia and Western Europe [4]. *B. distachyon* is closely related to many important cereals such as wheat, barley, rye, oats and forage grasses such as *Lolium* and *Festuca* [2,6,7], which makes it an excellent model for understanding the genetic, molecular and developmental biology of temperate grasses, cereals and dedicated biofuel crops such as switchgrass [3,6,8,9,10,11]. Similar to rice, wheat and barley, *B. distachyon* uses the C3 photosynthetic pathway [2,12]. In addition, it has many attributes that fit the characteristics of a model plant. Like Arabidopsis, *B. distachyon* has a small diploid genome (~310 Mb/1C), a small stature, a rapid life cycle, self-pollinates and has simple growth requirements [13]. Many tools and resources have been developed for *B. distachyon*, e.g., the complete genome sequence, a large collection of natural accessions, a high-density genetic map, genomic and cDNA libraries, an expressed sequence tag (EST) collections, microarrays, simple sequence repeat (SSR) markers, in vitro regeneration and T-DNA mutagenesis protocols [5,8,14,15]. As a model grass, *B. distachyon* has been used in biological studies, including those on root growth [16], stress tolerance [17,18], seed storage protein accumulation [19,20,21], fatty acid turnover [22], plant-pathogen interactions [23,24] and cell wall composition [11]. In addition, *B. distachyon* is amenable to in vitro manipulation and transformation.

Taking all these aspects into consideration, *B. distachyon* is a generally useful model in which to explore monocot biology. Despite this, the efficient transformation of *Brachypodium* species at a high frequency was, until very recently, a research bottleneck. In this review, we describe recent research and progress in in vitro *Brachypodium* research, which focus on genetic transformation, somatic embryogenesis, cell wall construction and reorganisation.

## 2. Refining the Transformation of *Brachypodium*

Genetic transformation introduces exogenous DNA into a recipient plant. The aim may be to create novel cultivars with improved properties or simply to test the function of a piece of DNA with known sequence. In both cases, the desired outcome is a fertile plant with a uniform, stable and ideally simple integration of the foreign DNA into the genome. Broadly, there are two ways to introduce DNA into a cell, either physical or via a biological vector. Commonly-used physical methods include ballistics where small particles are coated in DNA and shot into cells [25]. Vector mediated methods exploit a range of natural processes including viruses and pathogenic bacteria. In some species, such as Arabidopsis, protocols using the natural genetic engineer, *Agrobacterium*, allow efficient cost-effective transformation directly into the germ-line. In many other species, however, transformation requires plant regeneration in vitro from somatic tissues or cultured embryos. This in vitro process is not well understood, and efficiency of regeneration varies widely and can generate genetically unstable lines with altered chromosome number and copy number that varies from cell to cell [26,27].

Monocotyledonous plants tend to be more difficult to regenerate in in vitro culture [28,29]. Transformation is further limited by the recalcitrance of many genotypes to in vitro regeneration. The first transgenic wheat was obtained in 1992 by Vasil et al. [30], but barley was the last of the main cereal grains to be obtained as it quickly loses its ability to regenerate from an in vitro callus [31]. In rice, the *indica* genotype has remained recalcitrant to in vitro regeneration [32], which limits doubled haploid recovery. Moreover, the efficiency with which *Agrobacterium* transfers DNA to its “host” cell varies between and within species and, again, monocots tend to be less receptive.

However, over the last few years, there has been tremendous progress in the transformation of *Brachypodium,* which we describe below (Table 1).

### 2.1. Callus Induction

The first step in successful plant transformation is the ability to regenerate plants in tissue culture. The ability of grass species to be regenerated in vitro varies dramatically and is dependent on many factors: the age of the primary explantat, physiological state of the donor plant, genotype etc. [26]. Different species are amenable to transformation to different degrees and within each species there is differences between strains. For example, some grasses, such as *B. distachyon*, rice and wheat, have strains that are amenable to plantlet recovery from a culture and, therefore, can be transformed with reasonable efficiency. Other species such as oats and forage grasses are relatively lacking in such strains, which holds up progress on a number of fronts. The transformation protocols rely on the production of the embryogenic callus [8,36,37,45]. There is species-specific variation on the source of material used to produce embryogenic calli. In *Brachypodium,* while the most suitable source of explants for callus induction are immature embryos [3,14,33], calli with good efficiency have also been obtained from whole seeds [46,47]. Immature embryos of *B. distachyon* can be stimulated by an in vitro culture to re-enter the proliferative pathway and the first clusters of calli appear after one week of culture [48]. The calli that are derived from immature embryos are characterised by high regeneration potential and quality, which makes them the preferred target for genetic transformation [3,38]. Embryogenic calli are typically induced on an Murashige and Skoog medium (MS) or Linsmaier and Skoog Medium (LS) medium that has been supplemented with 2,4-dichlorophenoxyacetic acid (2,4-D) in different concentrations [33,38,46,48]. The optimal concentration of (2,4-D) for both callus induction and proliferation in *B. distachyon* is 2.5 mg l^−1^ [46,49]. Similar conditions can be used for other members of the genus, including *B. sylvaticum*, *B. stacei* and *B. hybridum* [42,48]. Although cell suspensions can also be generated, they require a higher concentration of 2,4-D (7.5 mg l^−1^) [50]. The embryos that were isolated from tetraploid accessions BDR017 and BDR030 had a greater potential to produce an embryogenic callus than those that had originated from the diploid accessions BDR001 and BDR018 [33], thus indicating that there could be a genetic variation for somatic embryogenesis as has been noted in some other grasses [32,51]. It should be noted that *Brachypodium* calli clearly age in a culture and have decreasing regenerative abilities [35,52]. A working compromise seems to be about six to nine weeks of culture, which allows the calli to increase in biomass to retain their embryogenic potential.

Although important advances have been made in our ability to regenerate *Brachypodium* embryogenic calli that are amenable to transformation, some challenges remain. While immature embryos tend to be the most commonly selected explants for callus induction because of their high transformation efficiency and regeneration capacity, they require a constant supply of healthy plants putting and thus access to controlled greenhouse conditions. Mature embryos can be stored (as seed) and are available throughout the year. Thus, a robust transformation protocol using mature embryos as the starting material would be very attractive. Indeed, mature embryos of three different *B. distachyon* accessions (BdTR4, BdTR6 and BdTR13) were successfully used as a plant material in both biolistic- and *Agrobacterium*-mediated transformation [53]. In addition to the source of material for callus induction, a number of parameters that benefit tissue culture have been suggested.

The next step in the transformation protocol is the delivery of the T-DNA to the regenerating cells. This can be done by different methods including particle bombardment, *Agrobacterium*-mediated transformation and protoplast transformation. We will describe each method and their advantages and disadvantages in the sections below.

### 2.2. Particle Bombardment

The first reported transformation [3] used particle bombardment on ABR100, an accession that has subsequently been re-classified as *B. hybridum* [54]. Since then, several studies have reported transforming embryogenic calli [46] with constructs carrying genes driven by different promoters and diverse selectable markers. The efficiency of transformation in these studies varied not only with the strain used but also with the culture conditions. Himuro et al. [43] suggested that growing Bd21 plants under a prolonged 20-h photoperiod yielded superior immature embryos that formed more proliferative and regenerative embryogenic calli than embryos that had been derived from seeds that had been developed under 16 h of light. Moreover, a transient osmotic treatment of the embryogenic calli enhanced the efficiency of the transformation via particle bombardment [33]. The time frame from the bombardment of an embryogenic callus to the harvesting of transgenic T1 seeds is about seven months. Since the seed-to-seed life cycle is 19 weeks, the *B. distachyon* transformation system permits both the T0 and the T1 generation as well as the production of T2 seeds to be tested within one year. Despite this, the biolistic method has fallen out of favour due to the complexity of the transgene loci, which can contain many copies, including truncated and rearranged sequences [55,56]. However, particle bombardment does not depend on the biological limitations of *Agrobacterium tumefaciens* and may be less dependent on the plant genotype.

### 2.3. Agrobacterium-Mediated Transformation

In contrast to biolistic transformation, *Agrobacterium*-mediated transformation typically results in a simple insertion pattern [57,58]. Although the development of an efficient *Agrobacterium*-mediated transformation systems for a monocot species can be difficult, *B. distachyon* is generally amenable and high-efficiency transformation methods have been developed [37,40,59]. A super-virulent strain of *Agrobacterium*, AGL1, and the *HPT* gene as a selective marker was used in 2006 [34] to transform ten *B. distachyon* lines (including Bd21). The average transformation efficiencies (expressed as the percentage of fertile transgenic plants per piece of calli) ranged from 0.2% to 13.5%. In this protocol, callus cultures were sub-cultured every two weeks by breaking them into smaller pieces (~2 mm diameter) and distributing them onto CIM. The callus was incubated in an *Agrobacterium* suspension for five minutes and then distributed onto a solid CIM medium that had been supplemented with acetosyringone and incubated for three days at 28 °C. Subsequently, the calli were transferred to a Timentin medium for one week in order to kill the *Agrobacterium* and then placed on a hygromycin medium and subcultured onto the same medium every two weeks. To regenerate plants, the surviving healthy calli were broken into pieces (~5 mm diameter) and placed on a regeneration medium. Both immature and mature seeds were used as explants for callus induction in order to avoid the laborious task of embryo isolation. While immature seeds tend to produce greater numbers of embryogenic calli, it should be noted that for some accessions, such as Bd11-1 and Bd13-1, an embryogenic callus was formed exclusively from mature seeds. The genetic basis of this variation is unknown but with the increasing availability of well-characterised biparental mapping populations and diversity panels, this gap could be closed.

An optimised transformation protocol for Bd21 was later developed and has become widely used. It included modifications such as a short (seven minute) drying of compact embryogenic calli (CEC) after they had been inoculated with *Agrobacterium* and the supplementation of the culture media with CuSO_4_. CEC can be recognised as dense regions within the more friable white calli, and visual selection of these enhance the chance of the recovery of successfully transformed plants. The calli were selected using hygromycin and then visually using a GFP reporter for approximately 17% of CEC-producing transgenic plants. To summarise this protocol: (1) immature embryos ≤0.3 mm were used to produce CEC, (2) a 3+2+1 subculture regime (which means callus cultivation for three weeks, then for two weeks and finally for one week, changing the medium to the new after each subculture) was used during the CEC production, (3) the CEC were inoculated with the *Agrobacterium* AGL1 strain (OD = 1.0) for five minutes followed by a seven-min drying treatment, (4) a combination of chemical selection (*HPT* gene) and visual screening (*GFP* gene) were used to rapidly identify the transgenic calli and plants and (5) the culture media was then supplemented with CuSO_4_ during callus induction and selection. On average, about 20 transformed lines were generated per 100 CECs used. Considering that one immature embryo produced ~40 CECs over six weeks, approximately eight transgenic plants could be produced from each immature embryo that enters the pipeline. Thole et al. [60] essentially used similar protocol to transform *B. distachyon* (Bd21), *B. hybridum* (PI 220567, Bd12-1) and *B. stacei* (TBd 8, ISK-P2). The highest transformation efficiencies were obtained for the ISK-P2 (20.1%) and Bd21 (20%) genotypes. An optimised protocol by Bragg et al. [39] had an average transformation efficiency of 42% and produced 8491 fertile T-DNA lines. A current *B. distachyon* transformation protocol, which is provided by the Vogel lab, is available at the JGI *B. distachyon* resource page [61]. An alternative protocol, which is based on Bd21-3 transformation, has also recently been published [62].

From these and other studies, several factors that affect transformation efficiency have emerged. The makeup of the vector plasmid that is used for the transformation can affect the transformation efficiency. A higher selection pressure, which is important for saving labour and time, was obtained using vectors with the maize ubiquitin promoter that drives the expression of the reporter genes than vectors with the CaMV 35S promoter [38,39]. Vectors that contain hygromycin selection genes are generally more efficient compared to those that use phosphinothricin selection. The genotype of the embryo also affects the efficiency of the pipeline, sometimes for relatively “trivial” reasons. The transformation efficiency was similar for both the Bd21-3 and Bd21 accessions, which are very closely related. However, the compact callus that was produced by Bd21-3 seemed to be more yellow and thus was easier to visually identify during culturing. There were also suggestions that the introduction of a second left border could limit the transfer of the vector DNA beyond the left border.

Similar methods can also be applied to other *Brachypodium* species. High transformation efficiency was observed (72%) when immature embryos from the perennial *Brachypodium sylvaticum* [42], accession PI269842, were used. As in many accessions of *B. distachyon*, only small immature embryos reliably generated an embryogenic callus. The best production of a callus occurred on an MS medium with maltose as the source of sugar and with added casein hydrolysate, as the source of vitamins and amino acids.

Completely avoiding tissue culture would, of course, be highly desirable. An *in planta* transformation procedure for *B. distachyon* [63] using the embryos of the Bd21-3 inbred line, which were pierced with a needle and then inoculated with *Agrobacterium,* has been described. This protocol is promising, but still requires further optimisation and can produce chimeric plants. Fursova et al. [41] co-cultivated mature trimmed *B. distachyon* seeds with an *A. tumefaciens* culture (EHA101 strain) for 30 h. The efficiency of the transformation was estimated to be around 5% of initially co-cultivated seeds.

Despite the recent improvements in the transformation efficiency and the range of species amenable to transformation, challenges still remain. The tissue culture process is still laborious and some of the embryogenic calli induction conditions need to be empirically determined for each new strain. As for now, *Agrobacterium*-mediated transformation is the most widely used process to generate transformed *Brachypodium* plants.

### 2.4. Protoplast-Based Assays

A fast functional characterisation of *B. distachyon* genes can also be obtained by transient gene expression in protoplasts. An efficient protocol for preparing leaf mesophyll protoplasts from *B. distachyon* seedlings as well as the polyethylene glycol (PEG)-mediated transformation procedure was presented in [64]. Two genes (*GFP* and *GUS*) were used as reporters to evaluate the feasibility of this transient expression system. The highest transient expression of the reporter genes was obtained when the protoplasts were transformed with 20 μg of plasmid DNA (10 min) and incubated for 16 h. The obtained results also showed that that plasmid size influences the transformation efficiency—smaller plasmids are more appropriate for use in transient expression assays.

Another protocol for the protoplast transformation of the inbred line Bd21-3, which was published in 2015 by Jung et al. [65], used young leaves from hydroponically grown Bd21-3 and determined that gene expression and/or protein localisation could be assessed between 24 h to 96 h later [66]. Although protoplast-mediated transformation can be very useful for initial functional tests and protein localisation studies, its transient nature limits its applicability. The ability to successfully regenerate *B. distachyon* plants from protoplasts could provide an important foundation for establishing a stable transformation.

## 3. Applications of *Brachypodium* Transformation in Functional Genetics

The transformation of *Brachypodium* species provides an effective route for transgenic plant production (reviewed in [2,40,67]) for functional genetics studies. Using T-DNA integration to modify gene expression is a highly effective tool for investigative studies, but ideally, a researcher should be able to find their favourite gene in a cost-effective manner. This requires gene-wide coverage with the reliable identification and cataloguing of the insertion sites. The adaptor-ligation PCR method enables the efficient retrieval of the flanking sequence tags (FSTs) of the T-DNA inserts [36,59]. An alternative method for obtaining the T-DNA flanking sequences is Inverse PCR (IPCR) [39]. T-DNA mutant lines are being developed by two research groups at the John Innes Centre (JIC) using genotype Bd21 [40,68] and by the U.S. Department of Agriculture Agricultural Research Center, Western Regional Research Center (USDA-ARS, WRRC) group using genotype Bd21-3 [39,69]. Unfortunately, the mutants that have been created by the JIC (~5000 lines) [40] are not easily accessible but requests can be directed to Germplasm Resources Information Network (GRIN) [70]. USDA-ARS, WRRC *B. distachyon* T-DNA mutant collection and ordering information are available at the Joint Genome Institute website [70], while the mapped insertion sites are available as a JBrowse track in the Phytozome database. An update (28.05.2019) indicated that 23,649 T-DNA *B. distachyon* lines are now available. The T-DNA mutants have mainly been created using vectors with the potential to create gene knockouts. However, some of the mutant collection comes from transformations with vectors that contain the “gene trap” sequences to infer the expression pattern of any disrupted genes and to identify promoters with tissue-specific expression patterns or transcriptional enhancers to overexpress nearby genes while maintaining normal expression patterns [39,69].

*B. distachyon* has contributed more widely to transgenic research in crops with many cereal T-DNA vectors including *Brachypodium* promoters [71]. Additionally, *B. distachyon* has been used in many functional studies that analyse plant development, stress responses and ion transport (Table 2).

## 4. Gene Editing in *Brachypodium* Species

Traditionally, plant transformation has been used to insert desirable T-DNA sequences or to perform gene disruption; however, in recent years, there have also been several reports of the successful editing of the *B. distachyon* genome using the Clustered Regularly Interspaced Short Palindromic Repeats (CRISPR/Cas9) targeted mutagenesis system or the Transcription activator-like effector nucleases (TALENs). The CRISPR/Cas9 technique relies on the cleavage of DNA by the Cas9 enzyme, which is guided to specific cleavage sites by guide RNAs [72]. Both the Cas9 enzyme and the guide RNAs are delivered into the plant cells together with the expression construct through transformation. The DNA repair mechanisms of plants will repair the Cas9-induced site-specific damage usually by the error-prone mechanism of non-homologous end joining and occasionally introduce desired DNA sequences at the cleavage site by the homology-directed repair. The TALEN technique leverages the artificial restriction enzymes that are generated by fusing a TAL effector DNA-binding domain to a DNA cleavage domain [73]. Transcription activator-like effectors (TALEs) can be quickly engineered to bind practically any desired DNA sequence.

As an example, eight B. distachyon genes, including BdABA1 (Bradi5g11750), BdCKX2 (Bradi2g06030), BdSMC6 (Bradi4g08527), BdSPL (Bradi2g03740), BdSBP (Bradi4g33770), BdCOI1 (Bradi2g23730), BdRHT (Bradi1g11090) and BdHTA1 (Bradi1g25390), were targeted using TALENs to generate knockout mutations [74]. TALEN-encoding constructs were introduced into the protoplasts using PEG transfection. Four TALENs had mutagenesis frequencies that ranged from 4% to 10%. The constructs were then introduced into B. distachyon embryonic cells using A. tumefaciens. Selection took place on a medium with hygromycin. The hygromycin-resistant callus lines were analysed for the presence of any mutations. Sixty-two mutant sequences were obtained and characterised as substitutions or small deletions or insertions. The frequencies of the TALEN-induced mutations in transgenic B. distachyon calli varied among the targeted genes from 5.9 to 100%. Unfortunately, the effect of the gene mutation on the phenotype of the mutants that were generated was not analysed.

The first successful use of CRISPR/Cas9 on *B. distachyon* was demonstrated by O’Connor [75]. The authors edited *Sister-of-PIN1* (*SoPIN1*, *Bradi4g26300*; the gene conserved in flowering plants), *PIN1a* (*Bradi1g45020*) and *PIN1b* (*Bradi3g59520*) genes using CRISPR. However, the researchers did not comment on the percentage of transformation success.

## 5. Factors That Affect the in Vitro Propagation of Grasses

The long-term maintenance of an in vitro culture is associated with a reduced regenerative capacity. For example, in vitro barley cultures quickly lose their ability to regenerate and tend to produce an increasing number of albino plants [96,97]. The molecular basis is completely obscure, but it may be due to cellular differentiation. Embryogenic cells have the typical features of undifferentiated cells such as a small or poorly developed vacuole, dense cytoplasm and a nucleus that usually has one and occasionally two nucleoli [98]. Mitoses are also occasionally present [99]. In contrast, non-embryogenic calli have parenchymatous or differentiated cells that are larger and highly vacuolated. Their nuclei are often inconspicuous and, if visible, they are usually located close to the cell walls.

In *B. distachyon*, older calli generally lose their embryogenic potential and endoploidy progressively increases after 90 days of cultivation [52]. The cell wall composition also changes. An embryogenic callus that was formed by cells that had been derived from the protodermal-dividing cells of the scutellum [98] initially had typical meristematic features and a high level of protein accumulation, which is connected with the acquisition of a competent state [100]. Embryogenic calli have an extracellular matrix on the surface of the calli cells (ECMSN), which is composed of the arabinogalactan proteins (AGPs) and pectins [99]. The pectins, AGPs and hemicelluloses can be used as molecular markers of the embryogenic cells. The arabinan-RG-I pectin-related epitope that is recognised by the LM16 antibody did not change during the culture period [9,99]. The pectic epitopes that are recognised by the LM19 and LM6 antibodies decreased, while the epitope that is recognised by the LM20 antibody increased over the course of the culture, which suggests that these pectins might be involved in the mechanisms that control the changes of cell fate during this process. The use of two monoclonal antibodies, JIM8 and JIM13, which recognise distinctive AGP epitopes, revealed in callus treated with 5-azacitidine (a potent hypomethylating agent) suggesting that these epitopes may be markers for cells which undergo cell death [101]. An immunohistochemical analysis revealed a decrease in the AGP signal over the course of the culture. Depending on the epitope, extensins (EXT) either increase (JIM12 epitope) or decrease (JIM11 epitope). Extensins may enhance the cell-cell contact within the calli, thereby promoting the transmission of the developmental signals. Clearly, the dynamics of the cell wall components changes along the timeline of a callus culture and this indicates that *B. distachyon* is a good model in which to dissect the regulation of cell differentiation. A large number of mutants and transgenics are available in which the cell wall properties are likely to be altered in defined ways, and therefore, it should be possible to assess whether the wall composition is important for regeneration. Similarly, natural accessions are known to vary in terms of their transformation and plantlet production [2,28]. The genetic basis of this variation should be dissected, which might reveal rate-limiting steps in grasses.

The characteristic metabolic profiles of *B. distachyon* tissue cultures have been associated with regenerative potential. Sixty- and 240-day-old calli had characteristic metabolic profiles including organic acids [102]. The profile of organic acids was correlated with both the growth and regenerative capacity of an aged callus and the decrease of metabolic activity over the course of 360 days. The organic acids may allow the production of endogenous amino acids to support the differentiation and morphogenetic activity; a non-morphogenic callus was found to have a very low content of the amino acids. It is likely that the regulatory genes are of even more interest and several somatic embryogenesis-related and cell cycle transcripts gradually decreased—*YUCCA* (*YUC*), *AINTEGUMENTA-LIKE* (*AIL*), *BABY BOOM* (*BBM*) and *CLAVATA* (*CLV3*) as well as for most of the cyclins—starting from the 30th day of a culture. Notably, the *WUSCHEL* (*WUS*) transcript was detectable only on the 30th and 60th days and was not detectable in the zygotic embryos or in the 90-day-old calli [52]. Many of these genes are related to the regulators of embryogenesis in Arabidopsis and can be rate-limiting for somatic embryogenesis in dicots [103]. If the situation is similar in grasses, it offers potential targets for enhancing somatic embryogenesis, and thereby, makes the biotech processes that depend on it much less expensive.

## Figures and Tables

**Table 1:** Reports on genetic transformation in *Brachypodium* species.

Genotype	Species	Target (explant)	Target Multiplication *	DNA Delivery	Marker Genes	Transformation Efficiency **	Overall Efficiency ***	Reference
**ABR100**	BH	Callus (IE)	NA	B	*HPT*, *GUS*	5 plant lines/g of bombarded tissue	NA	[3]
**BDR018** **BDR017** **BDR030**	BD BS BS	Callus (IE)	NA (6 weeks)	B	*BAR, GUS*	5.3% 3.8% 4.1%	NA	[33]
**Bd4-2** **Bd6-1** **Bd8-2** **Bd10-2** **Bd12-1** **Bd12-1** **Bd14-1** **Bd14-2** **Bd16-1** **Bd17-2** **Bd21**	BH BH BH BH BH BH BH BH BH BH BD	Callus (IS) Callus (IS) Callus (IS) Callus (IS) Callus (IS) Callus (MS) Callus (IS) Callus (IS) Callus (IS) Callus (IS) Callus (IS)	NA	Agro	*HPT, GUS*	0.2% 1.9% 0.4% 2.1% 2.1% 13% 0.2% 1.3% 1.5% 13.5% 3.2%	NA	[34]
**Bd21-3**	BD	Callus (IE)	×50 (6–7 weeks)	Agro	*HPT, GUS*	22.1%	11	[14]
**BDR018**	BD	Callus (IE)	×1 or less (17 days)	Agro	*BAR*, *GUS*	55%	0.6 or less	[35]
**Bd21Bd21-TC**	BD	Callus (IE)	×10 (4 weeks) ×16 or more (6 weeks)	Agro	*HPT*, *GFP*	5.1% 17%	0.5 2.7 or more	[36]
**Bd21**	BD	Callus (IE)	×40 (6 weeks)	Agro	*HPT, GFP*	20%	8	[37]
**Bd21**	BD	Callus (IE)	More than ×50 (7 weeks)	Agro	*HPT*, *GUS*	15.1%	More than 7.6	[38]
**Bd21-3**	BD	Callus (IE)	NA	Agro	*HPT*, *GUS*	42%	NA	[39]
**Bd21** **PI 220567** **Bd12-1** **TBd 8** **ISK-P2**	BD BH BH BS BS	Callus (IE)	×40 (6 weeks)	Agro	*HPT*	20% 12.2% 6.2% 12% 20.1%	8 4.9 2.5 4.8 8	[40]
**Bd21**	BD	MS	-	Agro	*GFP*	5%	0.1	[41]
**PI269842** **Ain-1**	BSYL BSYL	Callus (IE) Callus (IE)	NA	Agro	*HPT*, *GUS*	75% 22%	NA	[42]
**Bd21**	BD	Callus (IE)	NA	B	*HPT*, *GUS*	3.4%	NA	[43]
**Bd21** **Ain-1**	BD BSYL	Callus (IE) Callus (IE)	NA	Agro	*HPT* or *NPTII, GUS*	57.5% 4.3%	NA	[44]

Agro—*Agrobacterium*-mediated transformation, B—microparticle bombardment, *BAR*—*phosphinothricin acetyltransferase gene*, BD—*B. distachyon,* Bd21-TC—*Bd21* plants regenerated from tissue culture, BH—*B. hybridum*, BS—*B. stacei*, BSYL—*B. sylvaticum*, *GFP*—*green fluorescent protein gene*, *GUS*—*β-glucuronidase gene*, *HPT*—*hygromycin phosphotransferase* gene, IE—immature embryos, IS—immature seeds, MS—mature seeds, NA—not analysed, *NPTII*—*neomycin phosphotransferase II* gene. * Callus multiplication (at the date of the transformation) from a single immature embryo or seed (IS, MS) explant, ** Percentage of embryogenic calli (used as a target for transformation) that produced at least one transgenic plant, *** Number of independently transformed plant lines produced per original immature embryo (IE) or seed (IS, MS).

**Table 2:** Functional genomic studies using *Brachypodium* species transformation.

Genotype	Species	DNA Delivery	Gene	Encoded Feature/Characteristic	Major Findings	Reference
**Plant Growth and Development**						
**BDR017 BDR018**	BS BD	B	*LpTFL1* (from perennial ryegrass)*, TFL1* (from Arabidopsis)	Floral repressors	*LpTFL1* and *TFL1* transgenic plants had a significant delay of flowering	[76]
**Bd21**	BD	Agro	*eIF4A*	RNA helicase plays a key role in mRNA translation to protein	*eif4a* homozygous mutant plants were slow growing and had a reduced final plant stature	[77]
**Bd21**	BD	Agro	*BRI1*	Brassinosteroid receptor	*bri1* mutants had a dwarf and contorted plant phenotype and an altered epidermal cell shape and architecture	[40]
**Bd21**	BD	Agro	*AnGAL*, *AnAF*, *AnRAE* (from *Aspergillus nidulans*)	Hydrolases	The transient expression of the *A. nidulans* hydrolases affected the *B. distachyon* cell wall composition; transformed plants expressing *AnGAL* or *AnAF* had a reduced content of galactose and arabinose, respectively	[41]
**Bd21**	BD	Agro, P	*BdSOC1*	Transcription factor	Plants overproducing the truncated BdSOC1 forms had a delayed heading; truncated forms as well as heterodimers were mostly localised in the cytoplasm	[78]
**Bd21-3**	BD	Agro	*BdCESA4*, *BdCESA7*	Genes involved in cellulose biosynthesis	The *BdCESA4* and *BdCESA7* knock-down lines had a reduced stem area, the cell wall thickness of the xylem and fibres and the amount of crystalline cellulose in the cell wall	[79]
**Bd21** **Bd21-3**	BD	Agro	*BdTAR2L*	Gene involved in the auxin biosynthesis pathway	The *Bdtar2l* mutant had an elongated root phenotype	[80]
**Bd21-3**	BD	Agro	*BdEIN2L1*	Regulator of ethylene signalling	*bdein2l1* had an elongated root phenotype	[80]
**Bd21**	BD	Agro	*miR5200*	MicroRNA that is involved in the posttranscriptional regulation of the *FT* genes	Artificial interruption of the miR5200 activity accelerated the flowering time in short day (SD); miR5200 overexpression delayed flowering in a long day (LD)	[81]
**Bd21-3**	BD	Agro	*CAD*, *COMT*	Genes involved in lignin biosynthesis	Transgenic plants with a downregulated transcription of *BdCAD1* had a brown midrib phenotype; *BdCOMT4* downregulated plants had a reduced total lignin content	[82]
**Bd21-3**	BD	Agro	*FT1*	Gene involved in flowering induction	Shoots regenerated from the transgenic calli overexpressing *FT1* immediately developed floral organs under LD, none of the regenerated plants produced seeds; downregulation of *FT1* resulted in non-flowering *B. distachyon* plants	[83]
**Bd21-3**	BD	Agro	*VNR1*, *FT*	Genes involved in flowering induction	Plants overexpressing *VNR1* or *FT* had accelerated flowering without meeting the vernalisation requirement	[84]
**Bd21-3**	BD	Agro	*PHYC*	Light receptor	The flowering delay in the *phyC-1* mutants was compensated for by an overexpression of *FT*	[85]
**Bd21-3**	BD	Agro	*BdRGP1*	UDP-arabinopyranose mutase	The RNAi mutant of *BdRGP1* had a significant decrease of the cell wall arabinose content	[86]
**Bd21-3**	BD	Agro	*BdMUTE*	Transcription factor associated with guard mother cell (GMC) identity	The *Bdmute* mutants had dicot-like stomata, misoriented GMC divisions and aborted guard cells; the BdMUTE protein was mobile	[87]
**Bd21-3**	BD	Agro	*BdCESA8*	Cellulose synthase	*bdcesa8* mutants were dwarf, sterile and tended to fall over; xylem cells had irregular shapes	[69]
**Bd21-3**	BD	Agro	*BdCSLF6*	Mixed linkage glucan (MLG) synthase	The *bdcslf6* mutants had a reduced height and a decreased MLG content	[69]
**Bd21-3**	BD	Agro	*BdWAT1*	Tonoplast protein required for the proper formation of the secondary cell wall	The *bdwat1* plants were dwarf, developmentally delayed and had an irregularly shaped xylem	[69]
**Bd21-3**	BD	Agro	*BdRAD51*	Gene that is important for meiosis in both ovule and pollen development	The *bdrad51c* plants were vegetatively wild type but completely sterile; pollen was shrunken and deformed	[69]
**Bd21-3**	BD	Agro	*BdWAX2*	Gene that is important for cuticular wax biosynthesis	The *bdwax2* mutants had a decreased cuticular wax and lost their hydrophobicity	[69]
**Bd21-3**	BD	Agro	*BdTAB2*	Gene that is required for proper chloroplast function	The *bdtab2* mutants were yellow and died shortly after germination	[48]
**Bd21-3**	BD	Agro	*SoPIN1* *PIN1a* *PIN1b*	Auxin efflux carriers	The *sopin1* mutants had abnormal organ initiation during the flowering phase; *pin1a* and *pin1b* had an altered stem growth	[75]
**Bd21-3**	BD	Agro	*BdAUX1*	Auxin influx carrier	The *Bdaux1* mutants were dwarf, infertile and had an aberrant flower development and altered root phenotype	[88]
**Bd21-3**	BD	Agro	*FT2*	Gene that is involved in flowering induction	Overexpression of *FT2* resulted in precocious flowering and a reduced spikelet number	[89]
**Bd21** **Koz2** **Bd29-1**	BD	Agro	*FTL9*	Gene that is involved in flowering induction	Plants that contained an active *FTL9* allele had a SD-vernalisation response	[90]
**Bd21**	BD	Agro	*FTL9*	Gene an involved in flowering induction	The *ftl9* mutants growing under SD flowered significantly later than the wild-type plants, the overexpression of the *FLT9* gene significantly shortened the heading date under SD and led to a flowering delay under LD	[91]
**Abiotic and Biotic Stress Responses**						
**ABR1**	BD	B	*Bdpin1*	Proteinase inhibitor that is involved in wound- and jasmonate-mediated signalling	The promoter nature of the 5′ upstream region of *Bdpin1* was confirmed	[92]
**Bd21**	BD	B	*AtGolS2* (from Arabidopsis)	Enzyme that is involved in the biosynthesis of the raffinose family of oligosaccharides	The *AtGolS2*-expressing transgenic plants under drought stress showed less degreening than the wild-type plants and had a slight direct recovery following rehydration	[43]
**Bd21-3**	Bd	Agro	*AnAXE, AnRAE* (from *Aspergillus nidulans*)	Acetyl esterases	The AnAXE transgenic plants had a decreased degree of polysaccharide acetylation and an increased resistance to *Bipolaris sorokiniana*	[93]
**Bd21-3**	Bd	Agro P	*BdCBF1*	Transcription factors that are involved in the acclimation to low temperature process	Transgenic plants overexpressing the *BdCBF1* had an enhanced resistance to drought, salt and cold	[94]
**Other functions**						
**Bd21-3**	BD	P	*BdCOPT3* *BdCOPT4*	Genes that are involved in copper transport	BdCOPT3 and BdCOPT4 were localised in the plasma membrane	[66]
**Bd21-3**	BD	Agro	*1Dy10*	HMW-glutenin gene promoter	The expression of the wheat *1Dy10* gene promoter was observed only in the endosperm of mature seeds	[95]

Agro—*Agrobacterium*-mediated transformation, B—microparticle bombardment, BD—*B. distachyon*, BS—*B. stacei*, P—protoplast transformation.
